# Efficacy of triplet regimen antiemetic therapy for chemotherapy-induced nausea and vomiting (CINV) in bone and soft tissue sarcoma patients receiving highly emetogenic chemotherapy, and an efficacy comparison of single-shot palonosetron and consecutive-day granisetron for CINV in a randomized, single-blinded crossover study

**DOI:** 10.1002/cam4.373

**Published:** 2014-12-23

**Authors:** Hiroaki Kimura, Norio Yamamoto, Toshiharu Shirai, Hideji Nishida, Katsuhiro Hayashi, Yoshikazu Tanzawa, Akihiko Takeuchi, Kentaro Igarashi, Hiroyuki Inatani, Shingo Shimozaki, Takashi Kato, Yu Aoki, Takashi Higuchi, Hiroyuki Tsuchiya

**Affiliations:** Department of Orthopaedic Surgery, Graduate School of Medical Science, Kanazawa UniversityKanazawa, Ishikawa, 920-8641, Japan

**Keywords:** Antiemetic therapy, bone and soft tissue sarcoma, chemotherapy-induced nausea and vomiting, crossover trial, granisetron, palonosetron

## Abstract

The first aim of this study was to evaluate combination antiemetic therapy consisting of 5-HT_3_ receptor antagonists, neurokinin-1 receptor antagonists (NK-1RAs), and dexamethasone for multiple high emetogenic risk (HER) anticancer agents in bone and soft tissue sarcoma. The second aim was to compare the effectiveness of single-shot palonosetron and consecutive-day granisetron in a randomized, single-blinded crossover study. A single randomization method was used to assign eligible patients to the palonosetron or granisetron arm. Patients in the palonosetron arm received a palonosetron regimen during the first and third chemotherapy courses and a granisetron regimen during the second and fourth courses. All patients received NK-1RA and dexamethasone. Patients receiving the palonosetron regimen were administered 0.75 mg palonosetron on day 1, and patients receiving the granisetron regimen were administered 3 mg granisetron twice daily on days 1 through 5. All 24 patients in this study received at least 4 chemotherapy courses. A total of 96 courses of antiemetic therapy were evaluated. Overall, the complete response CR rate (no emetic episodes and no rescue medication use) was 34%, while the total control rate (a CR plus no nausea) was 7%. No significant differences were observed between single-shot palonosetron and consecutive-day granisetron. Antiemetic therapy with a 3-drug combination was not sufficient to control chemotherapy-induced nausea and vomiting (CINV) during chemotherapy with multiple HER agents for bone and soft tissue sarcoma. This study also demonstrated that consecutive-day granisetron was not inferior to single-shot palonosetron for treating CINV.

## Introduction

Chemotherapy-induced nausea and vomiting (CINV) is one of the most frequent nonhematologic toxicities associated with the treatment of malignant tumors. Symptoms such as CINV are a major cause of reduced quality of life in chemotherapy patients and lead to decreased therapy compliance. The frequency and timing of CINV manifestations differ according to the type, dose, and administration route of anticancer agents. Different types and dosage regimens of anticancer agents are categorized on the basis of the frequency with which they are associated with CINV: high emetogenic risk (HER) agents cause CINV in >90% of patients, moderate emetogenic risk agents cause CINV in 30% to 90% of patients, and low emetogenic risk agents cause CINV in <30% of patients.

Involvement of 5-HT_3_ receptors in CINV was reported in the late 1980s. During the 1990s, advancements were made in the clinical application of 5-HT_3_ receptor antagonists (5-HT_3_RA) for CINV prevention. The mechanism of CINV is thought to involve secretion of the neurotransmitter 5-hydroxytryptamine (serotonin) in response to stimulation of enterochromaffin cells in the gastrointestinal mucous membrane by anticancer agents. Transmission to the vomiting center then occurs either directly from the afferent vagal nerve via gastrointestinal 5-HT_3_ receptors or indirectly via the chemoreceptor trigger zone (CTZ). Another route involves direct stimulation of the CTZ by drugs, followed by transmission of this stimulus to the vomiting center via dopamine or 5-HT_3_ receptors 1. In addition to 5-HT_3_, recent evidence has suggested the involvement of the pain neurotransmitter substance P and its receptor neurokinin-1 (NK-1) in CINV. Aprepitant, an NK-1 receptor antagonist (NK-1RA), has been developed for clinical use 2. Corticosteroids also suppress CINV through an antiemetic effect that is thought to work primarily via anti-inflammatory action. A meta-analysis of clinical trials using antiemetic therapies, including dexamethasone (Dex), showed that the combination of 5-HT_3_RAs and Dex increased the control rates of acute and delayed nausea and vomiting by about 15% 3.

A meta-analysis of randomized controlled trials using antiemetic therapies showed that 5-HT_3_RAs have a superior antiemetic effect on the acute phase compared to conventional antiemetic therapies such as dopamine receptor antagonists and antihistamine drugs, and that 5-HT_3_RAs still play a central role in antiemetic therapy 4. However, problems with these agents remain. For instance, first-generation 5-HT_3_RAs do not exhibit a sufficient effect on delayed phase nausea and vomiting.

Palonosetron, a second-generation 5-HT_3_RA, has been shown to be highly selective for 5-HT_3_ receptors in a variety of experimental models. A clinical pharmacological study with healthy adult subjects showed that the half-life of palonosetron is 4–10 times longer than that of existing 5-HT_3_RAs. A long half-life and high affinity for 5-HT_3_ receptors are key characteristics of palonosetron. Also, chronological simulation of 5-HT_3_-receptor occupancy at recommended doses showed that a first-generation 5-HT_3_RA only maintained ≥70% receptor occupancy for <24 h, while the long half-life and high affinity of palonosetron allowed it to maintain this rate for about 5 days.

A multi-institution, randomized, blinded, controlled trial (the PROTECT study) compared single-shot intravenous palonosetron to single-shot intravenous granisetron (a first-generation 5-HT_3_RA) for controlling acute and delayed CINV 5. The subjects were patients with malignant tumors slated to receive at least 50 mg/m^3^ cisplatin (CDDP), doxorubicin and cyclophosphamide (AC), or epirubicin and cyclophosphamide (EC), which are all HER chemotherapy regimens. The patients received 0.75 mg palonosetron or 40 *μ*g/kg granisetron intravenously 30 min before the administration of chemotherapy. The acute complete response (CR) rate, which was the primary endpoint for effectiveness, was 75.3% in the palonosetron group and 73.3% in the granisetron group, demonstrating that palonosetron was not inferior to granisetron. However, the delayed CR rate was 56.8% in the palonosetron group and 44.5% in the granisetron group. This difference was significant, showing the superiority of palonosetron to granisetron. While this trial compared palonosetron and granisetron using single intravenous doses, consecutive-day administration of granisetron and other first-generation 5-HT_3_RAs has been approved and is used in patients receiving regimens that include consecutive-day administration of HER anticancer agents and multi-drug combinations.

Consecutive-day regimens involving multiple anticancer agents are widely used to treat bone and soft tissue sarcomas. Since many of these regimens use HER chemotherapy at high doses, treatment of bone and soft tissue sarcomas is frequently associated with CINV. Until recently, consecutive-day 5-HT_3_RA plus Dex was used for preventative antiemetic therapy; however, this regimen offers insufficient protection. Since the development of aprepitant, an NK-1RA, the combination of consecutive-day 5-HT_3_RA plus aprepitant and Dex, has become the recommended antiemetic therapy during chemotherapy for bone and soft tissue sarcomas. Although the PROTECT study found that single-shot palonosetron was superior to single-shot granisetron, there is no published comparative data on whether consecutive-day 5-HT_3_RA administration is superior to single-shot palonosetron.

The first aim of this study was to evaluate the efficacy of combination antiemetic therapy with a 5-HT_3_RA plus NK-1RA and Dex in patients receiving chemotherapy for high-grade bone and soft tissue sarcoma using multiple HER anticancer agents. The second aim was to compare the effectiveness and safety of single-shot palonosetron and consecutive-day granisetron in a randomized, single-blind, crossover trial.

## Materials and Methods

### Patients and treatment

Eligible patients were 15 years of age or older, had confirmed high-grade malignant bone and soft tissue tumors, and were scheduled to receive chemotherapy with multiple emetogenic anticancer drugs. Patients were required to have an Eastern Cooperative Oncology Group (ECOG) performance status of 0–2, as well as adequate bone marrow function (white blood cell count ≥2 × 10^3^ cells/L), hepatic function (aspartate aminotransferase and alanine aminotransferase <100 U/L), and renal function (creatinine clearance ≥60 mL/min). Exclusion criteria included any vomiting, retching, or grade ≥2 nausea according to the Common Terminology Criteria for Adverse Events (CTCAE), version 4, before administration of the study drug; known hypersensitivity to palonosetron, granisetron, other 5-HT_3_RAs, or Dex; participation in another drug study or receipt of any investigational agents within a month of study entry; and treatment with an antiemetic drug within the 24 h before administration of the study drug.

**Figure 1:**
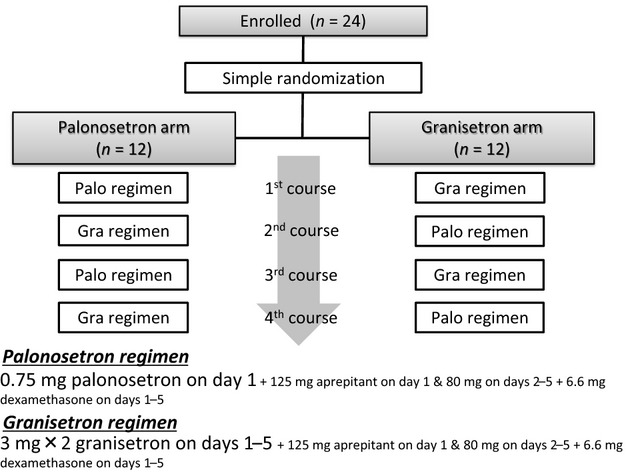
Study diagram.

A single randomization method was used to assign eligible patients to the palonosetron or granisetron arm. Patients in the palonosetron arm received antiemetic therapy with palonosetron during the first and third courses of chemotherapy, and granisetron during the second and fourth courses. Patients in the granisetron arm received granisetron during the first and third chemotherapy courses and palonosetron during the second and fourth courses (Fig.1). All patients were blinded to treatment assignment for the duration of the study.

For antiemetic therapy, all patients received 125 mg aprepitant (EMEND® Merck & Co., Inc., Whitehouse Station, NJ) orally 60 min before chemotherapy initiation and 6.6 mg Dex (DEXART injection®, Fuji Pharma Co., Ltd., Tokyo, Japan) intravenously 30 min before chemotherapy initiation on day 1. On days 2 through 5, 80 mg aprepitant and 6.6 mg Dex were sequentially administered. Additionally, patients receiving the palonosetron regimen were treated with 0.75 mg palonosetron (ALOXI®, Helsinn Healthcare SA, Pazzallo, Switzerland) intravenously 30 min before chemotherapy initiation on day 1. Patients receiving the granisetron regimen were treated with 3 mg granisetron (KYTTRIL®, F. Hoffmann-La Roche, Ltd., Basel, Switzerland) as a single fixed intravenous dose that was administered 30 min before chemotherapy initiation on day 1. Patients subsequently received 3 mg granisetron twice daily on days 1 through 4, and once daily on day 5 (Table1).

**Table 1:** Treatment regimens for antiemetic therapy

Treatment	Day 1	Days 2–4	Day 5
Palonosetron regimen	0.75 mg palonosetron	80 mg aprepitant	80 mg aprepitant
	125 mg aprepitant	6.6 mg dexamethasone	6.6 mg dexamethasone
	6.6 mg dexamethasone		
Granisetron regimen	3 mg × 2 granisetron	3 mg × 2 granisetron	3 mg × 1 granisetron
	125 mg aprepitant	80 mg aprepitant	80 mg aprepitant
	6.6 mg dexamethasone	6.6 mg dexamethasone	6.6 mg dexamethasone

Chemotherapy was administered at 3-week intervals. The chemotherapeutic regimens (Table2) were as follows: the AP regimen (120 mg/m^2^ CDDP [Randa®, Nippon Kayaku Co., Ltd., Tokyo, Japan] and 30 mg/m^2^ per day doxorubicin [Adriacin®, Kyowa Hakko Kirin Co., Ltd., Tokyo, Japan] for 2 days), the IE regimen (3 g/m^2^ per day ifosfamide [Ifomide®, Shionogi & Co., Ltd., Osaka, Japan] for 3 days and 60 mg/m^2^ per day etoposide [Lastet®, Nippon Kayaku Co., Ltd.] for 3 days), and the AI regimen (3 g/m^2^ per day ifosfamide for 3 days and 30 mg/m^2^ per day doxorubicin for 2 days). The regimen was chosen according to the pathological diagnosis and physical condition of the patient. The regimen could be changed after each course on the basis of the chemotherapeutic response and the degree of side effects associated with the treatment.

**Table 2:** Chemotherapy regimens

Treatment	Day 1	Day 2	Day 3
AP regimen	120 mg/m^2^ CDDP	30 mg/m^2^ DXR	
	30 mg/m^2^ DXR		
IE regimen	3 g/m^2^ IFO	3 g/m^2^ IFO	3 g/m^2^ IFO
	60 mg/m^2^ VP-16	60 mg/m^2^ VP-16	60 mg/m^2^ VP-16
AI regimen	3 g/m^2^ IFO	3 g/m^2^ IFO	3 g/m^2^ IFO
	30 mg/m^2^ DXR	30 mg/m^2^ DXR	

CDDP, cisplatin; DXR, doxorubicin; IFO, ifosfamide; VP-16, etoposide.

All participants were blinded to the antiemetic treatment assignments for the duration of the study. Patients were followed up for 10 days during each course for efficacy and safety endpoints. On days 4 (acute phase) and 10 (delayed phase), patients responded to a questionnaire that included questions about the number of emetic episodes, which were defined as single or multiple emetic experiences within short intervals regardless of the number of times a patient vomited. The use of rescue therapy, which included any medication taken to treat established nausea or emesis, was also recorded. Nausea severity was rated from 0 to 10 according to subjective assessment by each patient during the acute and delayed phases. After 4 courses of chemotherapy, patients were asked about their preferred regimen (even or odd regimen).

The primary endpoints of this study were the proportion of patients with CR (no emetic episodes and no rescue medication used) and total control (TC; no emetic episodes, no rescue medication used, and no nausea) during the overall phase (0–240 h post-chemotherapy), the acute phase (0–72 h post-chemotherapy), and the delayed phase (72–240 h post-chemotherapy), from 96 courses of chemotherapy in total. Secondary endpoints included CR and TC rates for the overall phase, acute phase, and delayed phase after the first course of chemotherapy and during courses 1 to 4 of chemotherapy; CR and TC rates for each chemotherapeutic regimen; the antiemetic regimen preferred by the patients; time to administration of rescue therapy; and severity of nausea.

This study was conducted in accordance with the Declaration of Helsinki, and written approval was obtained from the appropriate institutional review boards before the study commenced. All patients provided written informed consent before enrollment.

### Statistical analysis

Pearson's chi-squared (*χ*^2^) tests were applied as contingency table tests to investigate the associations between antiemetic regimens and preferred regimens. The difference in time to the administration of first rescue medication between the treatment groups was analyzed using Kaplan–Meier estimates, a log rank test, and the Cox proportional hazards model. Safety was assessed for all patients who received treatment. Safety data were tabulated and summarized descriptively. Toxicity grades were generated for hematology and blood chemistry parameters according to CTCAE-adapted toxicity grades, and treatment-related adverse events were tabulated. All p-values were two-sided, and the significance level was 5%. All statistical analyses were performed using SPSS software, version 19 (IBM Corp., Armonk, NY).

## Results

We enrolled 24 patients in this study between 1 April 2011, and 31 March 2013, and randomly assigned them to a palonosetron or granisetron arm. All patients received at least 4 courses of chemotherapy. All patients were eligible for efficacy analysis, and a total of 96 courses of antiemetic therapy were evaluated.

Demographic data are presented in Table3. For chemotherapy, the AP regimen was administered in 65 of 96 courses (68%), the IE regimen was administered in 20 courses (21%), and the AI regimen was administered in 11 courses (11%). Stratification showed the distributions of sex, chemotherapy regimen type, and ECOG performance status to be similar between the 2 groups. The most common types of malignancy were osteosarcoma (9 of 24 patients) and malignant fibrous histiocytoma (8 of 24 patients). Most patients (23 of 24 patients [92.9%]) had not received chemotherapy previously.

**Table 3:** Patient demographics and baseline characteristics

	Palonosetron arm	Granisetron arm	Total
Chemotherapy			
AP	33	32	65
IE or AI	15	16	31
Gender			
Male	7	5	12
Female	5	7	12
Age	36.1 (15–65)	50.6 (18–70)	43.4 (15–70)
ECOG performance status			
0	9	10	19
1	3	2	5
2	0	0	0
Tumor type	7 osteosarcoma	4 MFH	
	4 MFH	2 osteosarcoma	
	1 synovial sarcoma	2 leiomyosarcoma	
		1 rhabdomyosarcoma	
		1 DDLPS	
		1 MYLPS	
		1 clear cell sarcoma	

AI, ifosfamide plus doxorubicin; AP, cisplatin plus doxorubicin; DDLPS, dedifferentiated liposarcoma; ECOG, Eastern Cooperative Oncology Group; IE, ifosfamide plus etoposide; MFH, malignant fibrous histiocytoma; MYLPS, myxoid liposarcoma.

**Figure 2:**
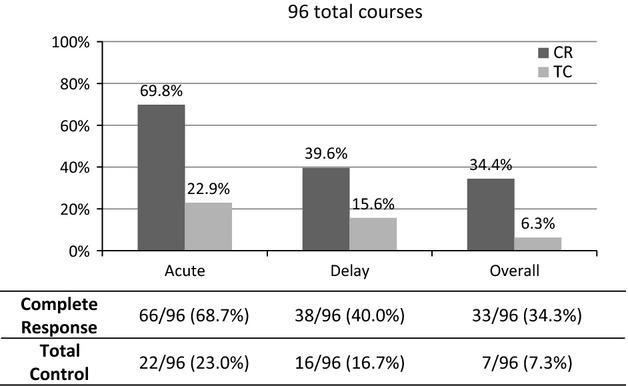
The overall chemotherapy-induced nausea and vomiting control rate with 5-HT_3_ receptor antagonists, a neurokinin-1 receptor antagonist, and dexamethasone in the 96 evaluated chemotherapy courses. The control rate in this study is clearly inferior to the control rates observed in other studies.

For the primary endpoint, a total of 96 courses of antiemetic therapy in 24 patients (4 courses per patient) were evaluated. The overall CR rate was 66 of 96 courses (69%) for the acute phase, 38 of 96 courses (40%) for the delayed phase, and 33 of 96 courses (34%) for the overall phase. The TC rates for the acute phase, delayed phase, and overall phase were 22 of 96 courses (23%), 16 of 96 courses (17%), and 7 of 96 courses (7%), respectively (Fig.2).

**Figure 3:**
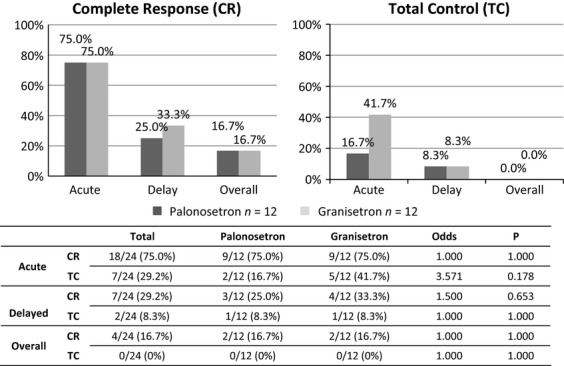
The percentage of patients (*n* = 24) who achieved a complete response (CR) and total control (TC) of chemotherapy-induced nausea and vomiting after the first course of chemotherapy during the acute phase (days 1–3 after chemotherapy initiation), the delayed phase (days 4–10), and overall phase (days 1–10). There were no significant differences in CR or TC between the palonosetron and granisetron regimens.

For the secondary endpoint, the first courses of chemotherapy in all 24 patients were evaluated. As first-course chemotherapy, 21 patients (87.5%) received AP chemotherapy and 3 patients (12.5%) received AI chemotherapy. Of the 21 patients treated with AP chemotherapy, 11 patients received the palonosetron antiemetic regimen, and 10 patients received granisetron. Of the 3 patients treated with AI chemotherapy, 1 patient received the palonosetron antiemetic regimen and 2 patients received granisetron. The CR rate during the acute phase (0–72 h) was 18 of 24 patients (75%) for both regimens, 9 of 12 patients (75%) for the palonosetron regimen, and 9 of 12 patients (75%) for the granisetron regimen. In the delayed phase (72–240 h), the CR rate was 7 of 24 patients (29%) for both regimens, 3 of 12 patients (25%) for the palonosetron regimen, and 4 of 12 patients (33%) for the granisetron regimen. The overall number of patients with CR for days 1 through 10 was 2 (17%) in both the palonosetron and granisetron regimens. TC was achieved by 7 of 24 patients (29%) in the acute phase; this included 2 of the 12 patients (17%) receiving palonosetron and 5 of the 12 patients (42%) receiving granisetron. In the delayed phase, 1 patient (8%) in both the palonosetron and granisetron groups achieved TC. The overall number of patients with TC from day 1 through 10 was 0 (0%). There was no significant difference in CR or TC between the regimens (Fig.3).

**Figure 4:**
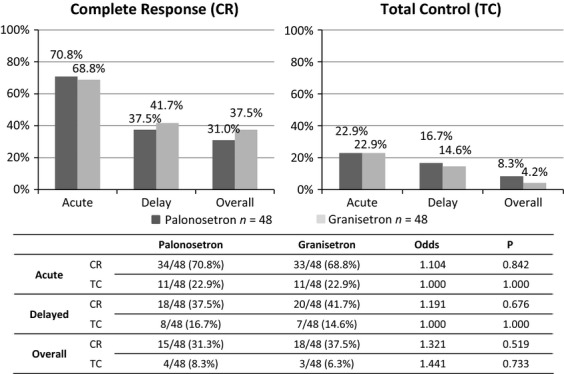
Comparative data for the palonosetron and granisetron regimens in the 96 evaluated chemotherapy courses. There were no significant differences in complete response or total control between the regimens in the acute, delayed, or overall phase.

Among a total of 96 courses of antiemetic therapy, 48 courses of palonosetron were administered with AP (33 courses), IE (10 courses), and AI (5 courses) chemotherapy. In addition, 48 courses of granisetron were administered with AP (32 courses), IE (10 courses), and AI (6 courses) chemotherapy. There were no significant differences between the chemotherapeutic agents used in the palonosetron and granisetron regimens. In the acute phase, CRs were achieved in 34 of 48 courses (71%) of the palonosetron regimen and in 33 of 48 courses (69%) of the granisetron regimen. In the delayed phase, CRs were achieved in 18 courses (38%) of the palonosetron regimen and in 20 courses (42%) of the granisetron regimen. The overall number of courses with CR for days 1 through 10 was 15 (31%) for the palonosetron regimen compared to 18 (38%) for the granisetron regimen. In the acute phase, TC was achieved in 11 of 48 (23%) courses for both the palonosetron and granisetron regimens. In the delayed phase, 8 courses (17%) of the palonosetron regimen and 7 courses (15%) of the granisetron regimen achieved TC. The overall number of courses with TC for days 1 through 10 was 4 (8%) on the palonosetron regimen and 3 (6%) on the granisetron regimen. There were no significant differences in CR or TC between the regimens (Fig.4).

**Figure 5:**
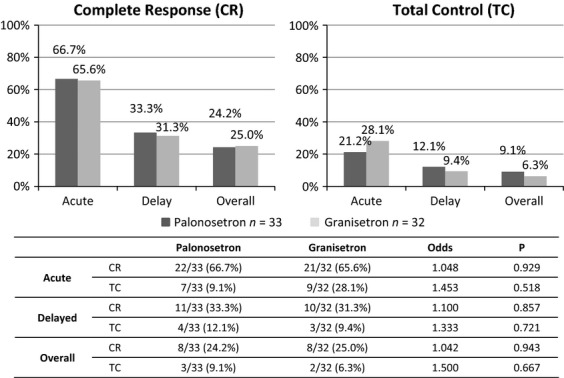
The chemotherapy-induced nausea and vomiting control rate for the cisplatin plus doxorubicin chemotherapy regimen. Thirty-three courses with palonosetron and 32 courses with granisetron were evaluated for antiemetic therapy.

**Figure 6:**
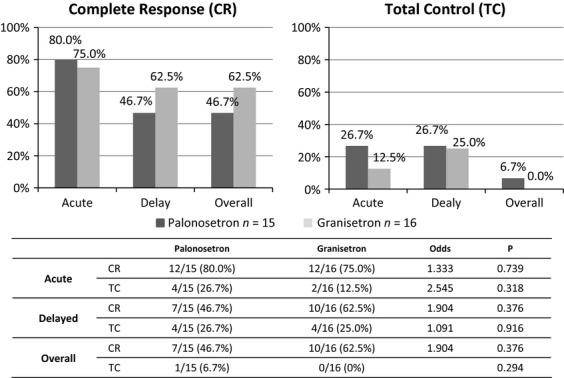
The chemotherapy-induced nausea and vomiting control rate for the ifosfamide plus etoposide and ifosfamide plus doxorubicin chemotherapy regimens. Fifteen courses with palonosetron and 16 courses with granisetron were evaluated for antiemetic therapy.

When each chemotherapeutic regimen was analyzed, there were no significant differences in CR and TC rates between the palonosetron and granisetron regimens; these data are shown in Figure5 (AP regimen, the most highly emetic regimen) and Figure6 (IE and AI regimens).

The patients who received the same chemotherapeutic regimen for 4 consecutive courses chose their preferred regimen after the fourth course of chemotherapy. Fifteen of the 24 patients were available for this evaluation. Two patients (13%) preferred the palonosetron regimen, 3 patients (20%) favored the granisetron regimen, and 10 patients (67%) replied that both the antiemetic regimens had similar efficacy. The number of patients who thought the regimens were equally effective was significantly larger than the number who favored a specific regimen (*P* = 0.022).

**Figure 7:**
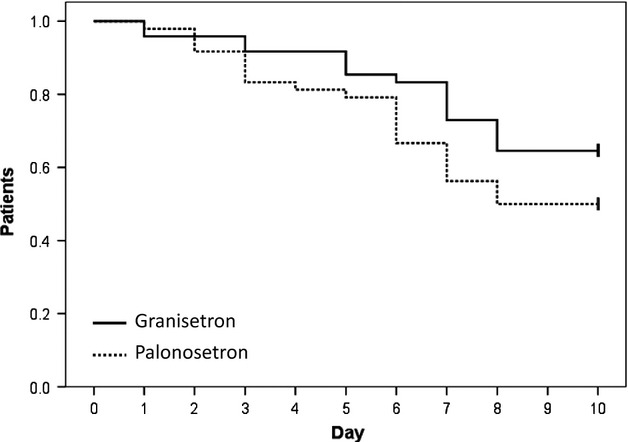
Kaplan–Meier curve of the time to first use of rescue medication. Tick marks at the ends of lines indicate patients who were censored for time-to-treatment failure at day 10. The time to first administration of rescue therapy tended to be longer for the granisetron regimen compared to the palonosetron regimen, but this difference was not significant.

The time to the first administration of rescue therapy tended to be longer for the granisetron regimen compared to the palonosetron regimen, but the difference was not significant (*P* = 0.115; hazard ratio = 1.610, 95% confidence interval = 0.864–2.999; Fig.7). For the palonosetron regimen, rescue therapy was administered in 24 of 48 courses, compared to 17 of 48 courses for the granisetron regimen. The median time to first use of rescue medication was 5.12 days for the palonosetron regimen and 5.65 days for the granisetron regimen.

**Figure 8:**
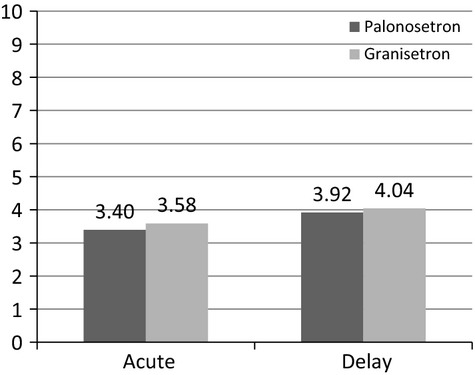
The average visual analog scale rating for nausea severity. There were no significant differences between the regimens in the acute or delayed phase.

Nausea severity was measured on a visual analog scale (VAS) from 0 to 10 according to subjective assessment during both the acute and delayed phases. The median VAS was slightly greater for the granisetron regimen than the palonosetron regimen in both the acute and delayed phases, though this difference was not statistically significant (acute phase: 3.40 for palonosetron versus 3.58 for granisetron; delayed phase: 3.92 for palonosetron vs. 4.04 for granisetron; Fig.8).

### Toxicity

Treatment-related toxicity is defined as a toxicity that occurs at or after the start of treatment that is possibly, or definitely, related to the treatment. All patients experienced at least 1 potentially treatment-related adverse event. Most of these adverse events were grade 1 or 2. Two patients experienced grade 3 toxicity on the palonosetron regimen: 1 patient had grade 3 stomatitis and 1 patient had grade 3 hiccups. There were no grade 4 toxicities.

A list of treatment-related adverse events for both treatment groups is provided in Table4. The most common treatment-related adverse event was constipation (49 of 96 total courses [51.0%]: 23 of 48 courses [47.9%] for palonosetron and 26 of 48 courses [54.2%] for granisetron), followed by increased serum aminotransferase concentrations (34 of 96 total courses [35.4%]: 17 of 48 courses [35.4%] for palonosetron and 17 of 48 courses [35.4%] for granisetron) and headaches (19 of 96 total courses [19.8%]: 12 of 48 courses [25.0%] for palonosetron and 7 of 48 courses [14.6%] for granisetron). There were no clinically relevant differences in the occurrence of adverse events between the antiemetic treatment regimens.

**Table 4:** Treatment-related adverse events occurring in at least 3 patients in 96 total courses of chemotherapy

	Palonosetron regimen *N* = 48			Granisetron regimen *N* = 48			Total *N* = 96		
	Grade 1	Grade 2	Grade 3	Grade 1	Grade 2	Grade 3	Grade 1	Grade 2	Grade 3
Constipation	15	8	0	19	7	0	34	15	0
Increased liver enzyme	13	3	0	14	3	0	27	6	0
Headache	12	0	0	6	1	0	18	1	0
Stomatitis	7	3	1	5	0	0	12	3	1
Hiccups	3	1	1	2	0	0	5	1	1
Dizziness	2	0	0	2	0	0	4	0	0
Taste disturbance	3	0	0	1	0	0	4	0	0
Diarrhea	3	2	0	1	2	0	4	4	0
Acne	2	1	0	1	0	0	3	1	0

## Discussion

Guidelines for antiemetic therapy during chemotherapy treatment have recently been proposed by several organizations including the American Society of Clinical Oncology 6, the National Comprehensive Cancer Network 7, the Multinational Association of Supportive Care in Cancer/European Society of Medical Oncology 8, and the Japan Society of Clinical Oncology 9. For HER chemotherapy, all guidelines recommend the combination of 5-HT_3_RA, NK-1RA, and Dex. For 5-HT_3_RA therapy, clinicians tend to recommend palonosetron on the basis of results from trials such as the PROTECT study.

However, for the consecutive-day regimens of anticancer agents used in this study, it is unclear which antiemetic agent should be administered and for what period. The regimen that includes CDDP (120 mg/m^2^ on day 1) plus doxorubicin (30 mg/m^2^ on day 1 and 2) is frequently used for sarcomas such as osteosarcoma. This regimen is classified as HER, but these highly emetogenic anticancer agents are used at higher doses than those administered in regular HER regimens. This regimen could be considered an extreme HER regimen, and it is unclear whether the regular antiemetic therapies described in the guidelines are capable of controlling the CINV associated with this regimen. In our current analysis, 3-drug antiemetic therapy achieved an acute CR rate of 69.8%, a delayed CR rate of 39.6%, and an overall CR rate of 34.4%. These CRs are clearly inferior to the rates achieved in other studies using the same 3-drug combination for HER chemotherapy regimens including ≥70 mg/m^2^ CDDP and AC therapy 10,11. In addition to differences in dosing and chemotherapy type, the relatively young age of our patients could explain the poor control rates we observed. Additionally, the antiemetic therapy guidelines are based on data from studies of typical moderately to highly emetogenic regimens; however, HER chemotherapy regimens administered for bone and soft tissue sarcomas may require stronger antiemetic therapies. Recent reports have suggested that adding olanzapine may be effective in cases where antiemetic therapy with the 3-drug combination does not achieve sufficient control 12,13. In the future, physicians should aim for even better CINV control by combining drugs, as described here.

Until now, no studies have compared the relative efficacy of single-shot palonosetron, which is often recommended by current guidelines, versus consecutive-day administration of 5-HT_3_RAs for extreme HER chemotherapy, such as the AP regimen. Therefore, we performed this comparison study based on the additional combination of NK-1RA and Dex. We did not find that palonosetron was superior for preventing delayed vomiting, which was observed by other studies. Consecutive-day administration of a first-generation 5-HT_3_RA is thought to have a similar effect on CINV as single-shot palonosetron, but examination of more cases is necessary to verify the statistical comparison of both drugs.

## Conclusions

Combination antiemetic therapy with 5-HT_3_RA plus NK-1RA and Dex did not effectively control CINV in patients with high-grade bone and soft tissue sarcoma treated with multiple HER chemotherapy regimens. Development of novel antiemetic agents, or new combination therapies with existing agents such as olanzapine, is needed.

This study demonstrated that consecutive-day granisetron administration was not inferior to single-shot palonosetron for controlling CINV associated with treatment of high-grade bone and soft tissue sarcoma using multiple HER chemotherapies.

## Conflict of Interest

None declared.
